# Abscesos cerebrales por *Nocardia* spp. en una paciente inmunocompetente

**DOI:** 10.7705/biomedica.4925

**Published:** 2020-03-30

**Authors:** Danilo E. Trujillo, Stephanie Ortiz, Óscar Pérez, Camilo A. Cortés, Jorge A. Carrillo

**Affiliations:** 1 Escuela de posgrados, Departamento de Medicina Interna, Universidad del Rosario, Bogotá, D.C., Colombia Universidad del Rosario Escuela de posgrados Departamento de Medicina Interna Universidad del Rosario BogotáD.C. Colombia; 2 Departamento de Medicina Interna, Fundación Cardioinfantil-Instituto de Cardiología, Bogotá, D.C., Colombia Fundación Cardioinfantil Instituto de Cardiología BogotáD.C. Colombia; 3 Área de Medicina Interna, Hospital Universitario Mayor - Méderi, Bogotá, D.C., Colombia Hospital Universitario Mayor - Méderi BogotáD.C. Colombia; 4 Área de Radiología e Imágenes Diagnósticas, Hospital Universitario Mayor - Méderi, Bogotá, D.C., Colombia Hospital Universitario Mayor - Méderi BogotáD.C. Colombia

**Keywords:** Nocardia, nocardiosis, absceso encefálico, antibacterianos, enfermedades cutáneas infecciosas, Nocardia, nocardia infections, brain abscess, anti-bacterial agents, skin diseases, infectious

## Abstract

La infección por *Nocardia* spp. no es común en pacientes inmunocompetentes. El tratamiento antimicrobiano empírico dirigido según las regiones anatómicas, no contempla las particularidades del germen y el análisis microbiológico se hace necesario para el tratamiento específico.

A continuación, se presenta el caso de una paciente previamente sana, inmunocompetente y sin factores de riesgo conocidos para la infección por *Nocardia* spp., con evidencia de compromiso en el parénquima pulmonar y la piel, que posteriormente desarrolló varios abscesos cerebrales.

## Abstract (en)

The infection by *Nocardia* spp is not common in immunocompetent patients. The empirical antimicrobial treatment directed by anatomical regions does not contemplate the particularities of the germ and the microbiological analysis is necessary for the specific treatment. We present the case of a previously healthy and immunocompetent patient, without known risk factors for *Nocardia* spp. infection, with evidence of involvement of the pulmonary parenchyma and the skin and subsequent development of multiple brain abscesses.

La nocardiosis es una condición rara en nuestro medio, causada por la infección por *Nocardia* spp., género de bacilos Gram positivos ramificados parcialmente y ácido-alcohol resistentes, el cual incluye más de 50 especies. La taxonomía de algunas de ellas aún se debate y, aproximadamente, la mitad se consideran relevantes debido a su capacidad de infección tanto en humanos como en animales. Estos bacilos son ubicuos en el ambiente y en casos muy aislados pueden estar presentes en la piel o en las vías respiratorias superiores ^1^. No se pueden considerar como microorganismos comensales y revisten importancia clínica en pacientes inmunocomprometidos, fundamentalmente aquellos con infección por el virus del HIV o linfoma, o con trasplante de órganos sólidos o de células madre hematopoyéticas, por lo que se le considera un agente patógeno oportunista. Se sabe que las personas con compromiso de la inmunidad celular están en riesgo de infección ^2^.

El uso de corticoesteroides en los pacientes con enfermedad pulmonar obstructiva crónica y enfermedades autoinmunitarias, también se ha asociado con el desarrollo de nocardiosis pulmonar y extrapulmonar ^3^. La infección pulmonar es la más común y el compromiso extrapulmonar es más frecuente en el sistema nervioso central. Hasta el momento, no existe una guía nacional o internacional de manejo para la nocardiosis y la mayoría de los datos corresponden a estudios descriptivos; en nuestro medio, se han descrito algunos casos de neuroinfección ^4^^-^^7^. Se han encontrado casos de nocardiosis en ausencia de factores de riesgo, por ello, ante el compromiso pulmonar o extrapulmonar, se debe sospechar la infección y considerar los estudios microbiológicos o moleculares adicionales para un adecuado diagnóstico.

A continuación, se describe el curso clínico de una paciente previamente sana que tuvo infección pulmonar sin que se le hubiera hecho aislamiento y en quien se diagnosticó nocardiosis extrapulmonar con abscesos en el sistema nervioso central.

## Descripción del caso

Se trata de una paciente de 50 años, mesera, y sin exposiciones de importancia a factores de riesgo, que consultó por un cuadro clínico de cinco semanas de evolución caracterizado por escalofríos, fiebre no cuantificada, astenia, adinamia y tos seca en accesos.

Inicialmente, se la trató en el servicio ambulatorio en un centro de atención primaria en donde se le tomó una radiografía simple de tórax que evidenció una consolidación incipiente en el lóbulo medio; fue tratada en casa durante siete días por vía oral con 750 mg de sultamicilina cada 12 horas y 500 mg de claritromicina cada 12 horas.

Dado que la sintomatología descrita persistió, a las dos semanas de culminado este tratamiento la paciente asistió a consulta en nuestra institución y allí se encontró que el tamaño de la consolidación en el lóbulo medio había aumentado y había leucocitosis con neutrofilia (leucocitos: 19,51 x 10^3^/µl, neutrófilos 15,27 x 10^3^/µl, linfocitos 2,15 x 10^3^/µl, monocitos 1,97 x 10^3^/µl, plaquetas 297 x 10^3^/µl), por lo que se decidió administrar por vía intravenosa 2 g de cefepime cada 8 horas durante siete días en el marco del programa de hospitalización en casa.

**Figura 1:**
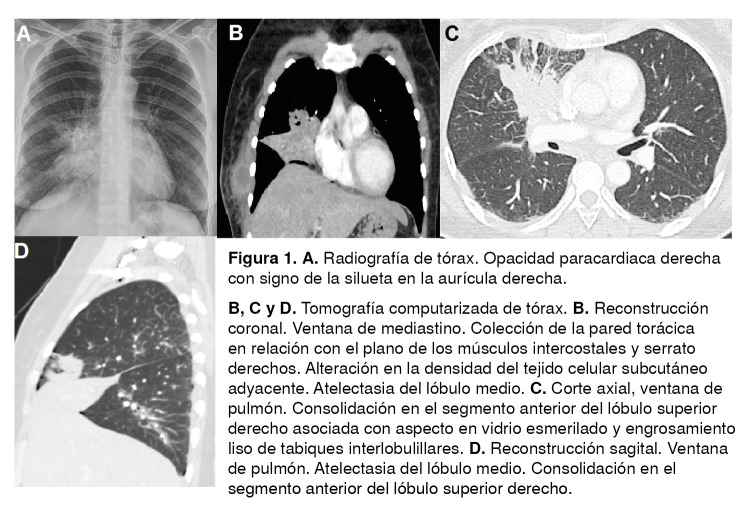
A. Radiografía de tórax. Opacidad paracardiaca derecha con signo de la silueta en la aurícula derecha. B, C y D. Tomografía computarizada de tórax. B. Reconstrucción coronal. Ventana de mediastino. Colección de la pared torácica en relación con el plano de los músculos intercostales y serrato derechos. Alteración en la densidad del tejido celular subcutáneo adyacente. Atelectasia del lóbulo medio. C. Corte axial, ventana de pulmón. Consolidación en el segmento anterior del lóbulo superior derecho asociada con aspecto en vidrio esmerilado y engrosamiento liso de tabiques interlobulillares. D. Reconstrucción sagital. Ventana de pulmón. Atelectasia del lóbulo medio. Consolidación en el segmento anterior del lóbulo superior derecho.

A pesar de la mejoría inicial (disminución de la tos y de la fiebre), la paciente volvió a consultar por presentar dolor en el hemitórax derecho y sensación de masa en la piel a nivel del sexto arco costal derecho. En el examen físico, se evidenció en la piel una formación sugestiva de absceso (eritematosa, bordes mal definidos, induración a la palpación, de 4 x 3 cm, aproximadamente) (figura 1), de la cual se obtuvo una muestra para cultivo, el cual fue negativo a los siete días de incubación.

Se intensificó el tratamiento, inicialmente con 1 g de meropenem cada 8 horas y 1,25 g de vancomicina cada 12 horas durante 14 días en el hospital. Culminado este esquema antibiótico, la paciente desarrolló cefalea intensa con signos de alarma, descrita como el peor dolor de cabeza de su vida, de instauración súbita, cuya intensidad aumentó progresivamente, y se acompañó de náuseas y emesis. La respuesta terapéutica al tratamiento analgésico con antiinflamatorios no esteroides por vía parenteral, acetaminofén por vía oral y tramadol, fue insuficiente.

**Figura 2:**
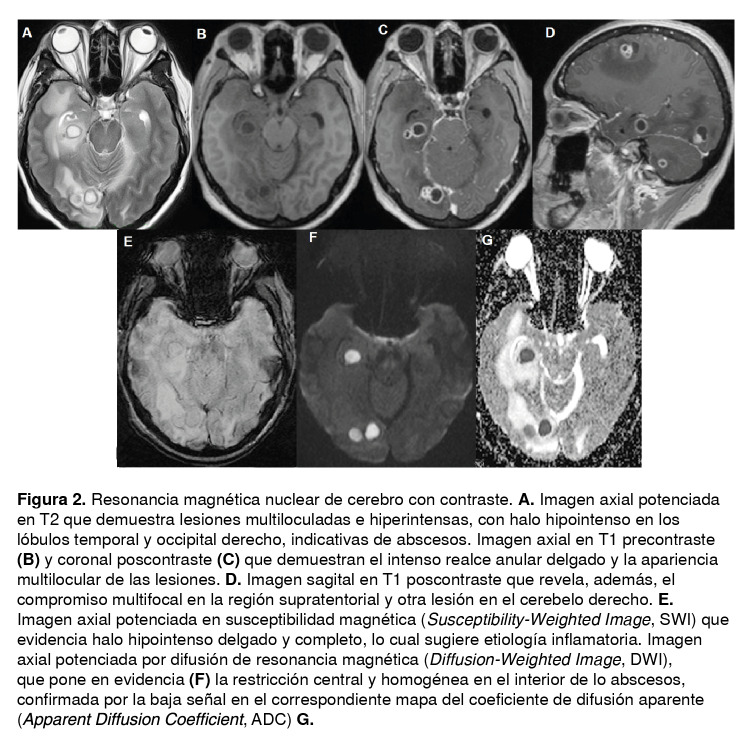
Resonancia magnética nuclear de cerebro con contraste. **A.** Imagen axial potenciada en T2 que demuestra lesiones multiloculadas e hiperintensas, con halo hipointenso en los lóbulos temporal y occipital derecho, indicativas de abscesos. Imagen axial en T1 precontraste **(B)** y coronal poscontraste **(C)** que demuestran el intenso realce anular delgado y la apariencia multilocular de las lesiones. **D.** Imagen sagital en T1 poscontraste que revela, además, el compromiso multifocal en la región supratentorial y otra lesión en el cerebelo derecho. **E.** Imagen axial potenciada en susceptibilidad magnética *(Susceptibility-Weighted Image,* SWI) que evidencia halo hipointenso delgado y completo, lo cual sugiere etiología inflamatoria. Imagen axial potenciada por difusión de resonancia magnética *(Diffusion-Weighted Image,* DWI), que pone en evidencia **(F)** la restricción central y homogénea en el interior de lo abscesos, confirmada por la baja señal en el correspondiente mapa del coeficiente de difusión aparente *(Apparent Diffusion Coefficient,* ADC) **G.**

La fiebre disminuyó hasta los 39 °C y el examen neurológico evidenció compromiso del tercer par craneano derecho, sin signos de irritación meníngea u otras alteraciones, por lo que se le practicó una resonancia magnética cerebral simple con contraste, la cual evidenció múltiples lesiones en el parénquima cerebral con edema vasogénico asociado (figura 2).

Se descartaron: infección por HIV (ELISA de cuarta generación con resultado no reactivo); tuberculosis (baciloscopias seriadas en tres momentos diferentes, todas negativas, así como cultivos para micobacterias en medio líquido a partir de muestras de esputo, lavado broncoalveolar y piel, con resultados negativos); neoplasias gastrointestinales y abdominales (esofagogastroduodenoscopia y colonoscopia con biopsias negativas para malignidad y atipia celular, tomografías con contraste de tórax y abdomen en las que se evidenció disminución significativa de la consolidación pulmonar, resolución de la lesión en tejidos blandos y la piel del hemitórax derecho sin otros hallazgos relevantes); infección por virus linfotrópicos (pruebas de IgM e IgG no reactivas para citomegalovirus y virus de Epstein-Barr), y hepatotrópicos (pruebas no reactivas de anticuerpos para virus de hepatitis C, antígeno de superficie de hepatitis B, anticuerpos para antígeno de superficie de hepatitis B, anticuerpos anti-core totales de hepatitis B y anticuerpos IgM para hepatitis A). Se practicaron una fibrobroncoscopia y un lavado broncoalveolar en el que se registró un conteo elevado de neutrófilos (18 %) con cultivo negativo para hongos, bacterias y micobacterias, en tanto que la citometría de flujo del líquido cefalorraquídeo no arrojó resultados sugestivos de un proceso infeccioso o neoplásico.

Ante dichos resultados y dada la persistencia de la cefalea, la fiebre y el compromiso del tercer par craneano derecho, se tomó una biopsia de una de las lesiones cerebrales y se sometió a estereotaxia, con lo cual se halló infiltrado inflamatorio; el cultivo fue positivo para *Nocardia* spp. a los tres días de incubación. Se inició el tratamiento antibiótico con trimetoprim-sulfametoxazol (300 mg/1.500 mg cada 8 horas) y 1 g de ceftriaxona por vía intravenosa cada 12 horas, el cual fue tolerado adecuadamente por la paciente y tuvo mejoría clínica: disminución progresiva de la cefalea, ausencia de fiebre, resolución de leucocitosis y mejoría de las lesiones evidenciada en la resonancia magnética nuclear cerebral de control.

La paciente fue valorada en oftalmología y no se evidenció compromiso ocular. El cuadro hemático y el frotis de sangre periférica estuvieron dentro de límites normales sin atipia celular evidente, y los anticuerpos antinucleares y el factor reumatoide fueron negativos. Se continuó el tratamiento con trimetoprim-sulfametoxazol y ceftriaxona a la dosis descrita y completó dos meses; se administraron seis meses más de trimetoprim-sulfametoxazol en el servicio de hospitalización en casa.

El control con resonancia magnética a los siete meses del tratamiento con trimetoprim-sulfametoxazol evidenció una disminución del número de las lesiones, así como de la reacción inflamatoria en torno a estas. Por ello, en el momento de la elaboración de este reporte, se cambió la administración de trimetoprim-sulfametoxazol a la vía oral y una dosis de 320 mg/1.600 mg cada 8 horas, para completar 12 meses de tratamiento.

## Consideraciones éticas

Para la publicación de este caso se contó con el consentimiento informado de la paciente, ajustado al protocolo institucional. Se preservan el nombre y los datos de identificación para proteger la privacidad de la paciente.

## Discusión

La aparición de nocardiosis en un paciente inmunocompetente es rara, por lo que se debe descartar la inmunosupresión por cualquier causa. La presentación más común de esta infección se caracteriza por la presencia de neumonía necrosante asociada con cavitación, en la que las bronquiectasias o alteraciones estructurales del parénquima pulmonar son un factor de riesgo conocido para la colonización pulmonar ^8^.

No obstante, en 33 a 44 % de los casos existe compromiso extrapulmonar, principalmente del sistema nervioso central, secundario a la diseminación hematógena. Los síntomas dependen de las estructuras comprometidas (meninges, corteza, cerebelo o estructuras vasculares) y la principal manifestación es la formación de abscesos ^1^^,^^3^.

Además, la inoculación directa del microorganismo en la piel puede desencadenar nocardiosis cutánea primaria que, en los huéspedes inmunocompetentes, frecuentemente se asocia con la exposición a factores de riesgo. Si bien en la mayoría de los casos estudiados hasta el momento, la puerta de entrada de la infección son las vías respiratorias, en el caso de esta paciente, los aislamientos microbiológicos de secreción respiratoria y líquido pleural no permitieron confirmar esta asociación.

La diferenciación entre las especies es difícil y la evaluación de los patrones de resistencia es importante a la hora de determinar el tratamiento antibiótico; sin embargo, hasta el momento no hay un esquema validado a nivel local o internacional ^4^. Para la detección y la tipificación, se usa una variedad de pruebas moleculares con nuevas tecnologías, lo que aumenta la posibilidad de aislamiento ^9^.

La paciente de este caso no tenía antecedentes de exposición a los factores de riesgo conocidos para nocardiosis. Se presumió el compromiso pulmonar, así como en la piel y el sistema nervioso central; sin embargo, solo se logró el aislamiento con la biopsia sometida a estereotaxis de una de las lesiones del sistema nervioso central sin necesidad de recurrir a pruebas moleculares. La lesión en la piel era un absceso, lo cual coincide con lo descrito en la literatura médica como resultado de la inoculación directa, que puede causar abscesos y celulitis localizada semejantes a los de la infección por *Staphylococcus aureus* o estreptococos. Se conoce un caso de nocardiosis con varios abscesos cerebrales en un paciente inmunocompetente internado en el Hospital Universitario Mayor - Méderi, en quien se aisló mediante técnicas moleculares *Nocardia beijingensis* y que finalmente falleció después de presentar compromiso multisistémico ^10^.

La búsqueda activa de la etiología de las lesiones pulmonares en la paciente, además del curso clínico descrito, permitió la evaluación diagnóstica inicial de posibles condiciones inmunosupresoras a nivel celular (enfermedad neoplásica sólida o hematológica o autoinmunitaria, tratamiento con corticoides, etc.), las cuales constituyen factores de riesgo para el desarrollo de esta enfermedad. Debe anotarse, no obstante, que se han reportado casos similares de nocardiosis en pacientes inmunocompetentes ^11^^,^^12^.

En cuanto al tratamiento, el inicio de la administración de dos o tres antibióticos depende del estado general del paciente y del curso clínico de la infección, ya que no hay suficientes estudios que validen su uso, aunque el esquema triple se justifica en pacientes con infección grave.

La combinación de trimetoprim-sulfametoxazol, amikacina y ceftriaxona o carbapenémicos, ha sido la de elección en casos graves de nocardiosis ^13^. Debe considerarse, además, la adecuada penetración de antibióticos como el trimetoprim-sulfametoxazol y la ceftriaxona por vía endovenosa en el sistema nervioso central y la evolución del paciente, para determinar el momento apropiado de cambio al esquema oral. Entre los carbapenémicos, el imipenem ha sido el más seleccionado en este contexto, pero según estudios moleculares, el ertapenem y el meropenem son opciones igualmente válidas ^14^^,^^15^.

La duración del tratamiento se orienta a disminuir el riesgo de recaída de la enfermedad, por lo que se debe mantener por un tiempo prolongado; en el caso de pacientes inmunocompetentes con infección pulmonar o sin compromiso del sistema nervioso central, un esquema de 6 a 12 meses se puede considerar apropiado. Sin embargo, cuando se detecta *Nocardia* spp. a nivel cerebral, como en el caso de esta paciente, se debe completar un tiempo mínimo de un año. En cuanto a los abscesos cerebrales que forman lesiones multiloculares de paredes gruesas, existe la posibilidad del manejo quirúrgico como tratamiento adyuvante según el curso clínico ^16^.

## Conclusión

La nocardiosis es una enfermedad infrecuente en pacientes inmunocompetentes cuyo diagnóstico siempre requiere los estudios microbiológicos para administrar el tratamiento dirigido, ya que las manifestaciones clínicas no permiten la diferenciación adecuada para iniciar el manejo empírico más acertado. Si hay antecedentes de riesgo para la infección por *Nocardia* spp., se debe sospechar su presencia. El tratamiento empírico debería cubrir las particularidades del germen, pero aún no hay información comprobada que permita establecer una ruta para su manejo y su estudio.

El diagnóstico temprano de nocardiosis es difícil dadas las limitaciones para su aislamiento; además, se desconoce si hay estudios de costo-efectividad de las pruebas moleculares en este contexto, por lo que se carece de herramientas para determinar su utilidad frente a los estudios microbiológicos.
